# Visualized effect of oxidation on magnetic recording fidelity in pseudo-single-domain magnetite particles

**DOI:** 10.1038/ncomms6154

**Published:** 2014-10-10

**Authors:** Trevor P. Almeida, Takeshi Kasama, Adrian R. Muxworthy, Wyn Williams, Lesleis Nagy, Thomas W. Hansen, Paul D. Brown, Rafal E. Dunin-Borkowski

**Affiliations:** 1 Department of Earth Science and Engineering, Imperial College London, South Kensington Campus, London SW7 2AZ, UK; 2 Center for Electron Nanoscopy, Technical University of Denmark, Kongens Lyngby DK-2800, Denmark; 3 School of GeoSciences, University of Edinburgh, The King’s Buildings, West Mains Road, Edinburgh EH9 3JW, UK; 4 Division of Materials, Mechanics and Structures, Department of Mechanical, Materials and Manufacturing Engineering, Faculty of Engineering, University of Nottingham, University Park, Nottingham NG7 2RD, UK; 5 Ernst Ruska-Centre for Microscopy and Spectroscopy with Electrons and Peter Grünberg Institute, Forschungszentrum Jülich, Jülich D-52425, Germany

## Abstract

Magnetite (Fe_3_O_4_) is an important magnetic mineral to Earth scientists, as it carries the dominant magnetic signature in rocks, and the understanding of its magnetic recording fidelity provides a critical tool in the field of palaeomagnetism. However, reliable interpretation of the recording fidelity of Fe_3_O_4_ particles is greatly diminished over time by progressive oxidation to less magnetic iron oxides, such as maghemite (γ-Fe_2_O_3_), with consequent alteration of remanent magnetization potentially having important geological significance. Here we use the complementary techniques of environmental transmission electron microscopy and off-axis electron holography to induce and visualize the effects of oxidation on the magnetization of individual nanoscale Fe_3_O_4_ particles as they transform towards γ-Fe_2_O_3_. Magnetic induction maps demonstrate a change in both strength and direction of remanent magnetization within Fe_3_O_4_ particles in the size range dominant in rocks, confirming that oxidation can modify the original stored magnetic information.

## Abstract (web summary)

Magnetite provides a valuable record of the Earth's geomagnetic history. Here, Almeida *et al*. combine electron microscopy and energy-loss spectroscopy to study the effects of *in situ* oxidation on the magnetization fidelity and crystalline phase of pseudo-single domain magnetite grains.

## Graphical abstract

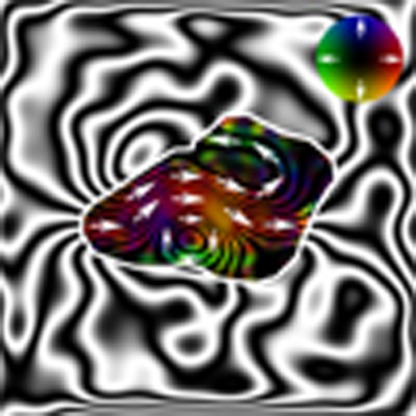

Magnetic minerals in rocks record the direction and intensity of the Earth’s ambient magnetic field during formation, providing, for example, information on past tectonic plate motion and the evolution of the geodynamo. In order to reliably interpret palaeomagnetic data, the mechanisms that induce and influence magnetic remanence within rocks must be fully understood. Some mechanisms such as thermoremanent magnetization are broadly understood^1^; however, the wide class of mechanisms that come under the heading of chemical remanent magnetization (CRM) requires more detailed study. Current models for CRM processes are, with one exception^2^, presently restricted to small, uniformly magnetized, single-domain (SD) grains^3^. Models for other types of chemical alterations are at best phenomenological^4^, and CRM processes in larger grains containing nonuniform magnetization states (that is, multidomain (MD) states) are little understood. Nevertheless, magnetic signals from rocks are often dominated by small MD grains that exhibit magnetic recording fidelities similar to those of SD grains (termed pseudo-SD (PSD)). For PSD grains, there are two key palaeomagnetic questions: does chemical alteration affect the directional record? To what extent does chemical alteration affect magnetization intensity?

Magnetite (Fe_3_O_4_) is probably the most important magnetic mineral on Earth because of its high abundance and strong, dominating magnetization. However, its ability to preserve the remanence of the Earth's magnetic field is greatly influenced by progressive oxidation, at ambient pressures and temperatures, to less magnetic iron oxides such as maghemite (γ-Fe_2_O_3_) or haematite (α-Fe_2_O_3_). Hence, oxidation of PSD Fe_3_O_4_ grains is a critical component of the CRM process and must be fully understood to allow for reliable interpretation of palaeomagnetic data.

Off-axis electron holography in the transmission electron microscope (TEM) allows nanometre-scale imaging of magnetic induction within and around materials as a function of applied field and temperature^5^,^6^,^7^,^8^,^9^. For example, Feinberg *et al*.^10^ used off-axis electron holography to show how the un-mixing of a titanomagnetite inclusion within a natural clinopyroxene matrix resulted in the development of an internal microstructure consisting of Fe_3_O_4_ prisms and ulvöspinel Fe_2_TiO_4_ lamellae, transforming it from an individual MD grain structure into an assemblage of magnetostatically interacting SD prisms. In particular, it was shown that the overall remanence direction was dependent on both the inclusion’s elongation direction and the prism arrangements therein^10^, thereby demonstrating the ability of chemical alterations to affect the magnetic remanence of natural magnetic recorders.

To fully appraise the effects of chemical alterations on CRM processes, it becomes necessary to investigate changes of magnetic domain structures in grains, directly, under controlled conditions. In this context, we present the application of a range of complementary electron microscopy techniques to examine local changes in the magnetization of PSD Fe_3_O_4_ grains, as they chemically alter during *in situ* heating within a controlled oxidizing atmosphere. The technique of environmental TEM (ETEM), combined with spherical aberration (C_S_) correction, enables localized chemical reactions under gas atmospheres to be observed with an interpretable spatial resolution on the sub-ångström scale^11^, while complementary electron energy-loss spectroscopy (EELS) investigations allow changes in material oxidation state to be appraised^12^. Off-axis electron holography enables nanometre-scale imaging of magnetic induction within and around magnetic grains, as a function of oxidation, through the generation of magnetic induction maps.

We show here the combined use of ETEM and off-axis electron holography to directly visualize the effect of oxidation on the recording fidelity of individual PSD Fe_3_O_4_ grains. Construction of magnetic induction maps demonstrates a change in both strength and direction of remanent magnetic states within the Fe_3_O_4_ grains, as a consequence of oxidation, and we discuss how it can lead to an underestimation of the ancient geomagnetic field strength and the geodynamo magnetic moment.

## Results

### Effect of oxidation on strength of magnetization

**Figure 1:**
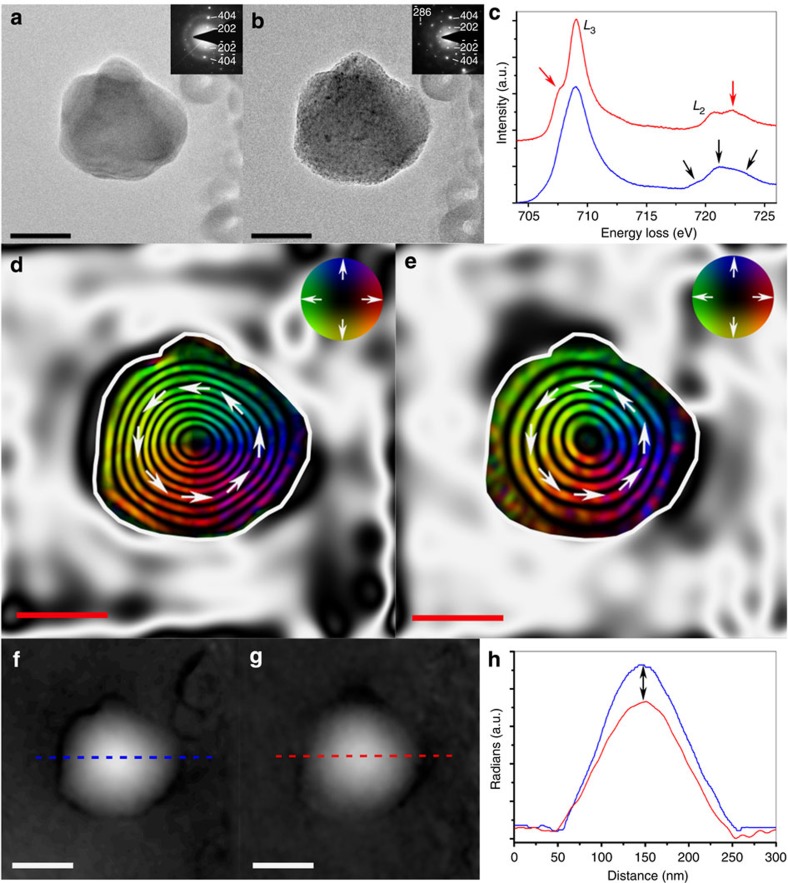
Visualized effect of oxidation on the magnetization of an equiaxed Fe_3_O_4_ particle. Bright-field TEM images acquired (**a**) before and (**b**) after *in situ* heating to 700 °C under 9 mbar of O_2_ for 8 h in an ETEM, with associated SAED patterns inset, indexed to Fe_3_O_4_ (Joint Committee on Powder Diffraction Standards (JCPDS) No. 75–449). (**c**) Associated EEL spectra of the Fe 2*p L*_2,3_ edge acquired from the Fe_3_O_4_ particle before (blue) and after (red) annealing within the ETEM. Black arrows emphasize three differing intensities from the mixed-valence compound of Fe_3_O_4_, while the red arrows highlight formation of pre- and post-peaks that indicate oxidation towards γ-Fe_2_O_3_. (**d**,**e**) Magnetic induction maps determined from the magnetic contribution to the phase shift, reconstructed from holograms taken (**d**) before and (**e**) after *in situ* heating, revealing the vortex nature of the particle. The contour spacing is 0.79 radians for both magnetic induction maps. The magnetization direction is shown using arrows, as depicted in the colour wheel. (**f**,**g**) Magnetic contributions to the phase shift, as used to reconstruct the magnetic induction maps in (**d**,**e**), respectively, and (**h**) line profiles across their centers before (blue) and after (red) annealing. Black arrows in **h** illustrate the loss in overall magnetic remanence. Scale bars represent 100 nm.

Figure 1 illustrates the effect of accelerated oxidation on the magnetization of an individual, equiaxed synthetic Fe_3_O_4_ grain, as assessed using TEM, selected area electron diffraction (SAED) and EELS, along with associated magnetic induction maps. The bright-field TEM image of Fig. 1a shows a native, smooth-surfaced, ~200 nm diameter Fe_3_O_4_ grain, as indicated by SAED (Fig. 1a, inset). EELS analysis of the Fe 2*p L*_2,3_ edge, in the region 704–726 eV (Fig. 1c), confirmed the assignment of pure Fe_3_O_4_. The *L*_2_ edge for this sample shows the typical shape of a mixed-valence compound, that is, three visible features of differing intensities (Fig. 1c, black arrows), while the almost-shapeless *L*_*3*_ edge is attributed to the combined spectral contributions of different iron sites (that is, Fe^2+^ at octahedral B-sites and Fe^3+^ at both tetrahedral A and octahedral B-sites), consistent with the more delocalized structure of Fe_3_O_4_, as compared with other mixed iron oxides^13^,^14^. The corresponding magnetic induction map of Fig. 1d exhibits evenly spaced magnetic contours, spanning from the surface to the centre of the grain, flowing in a counterclockwise direction (denoted by arrows), characteristic of a vortex state.

**Figure 2:**
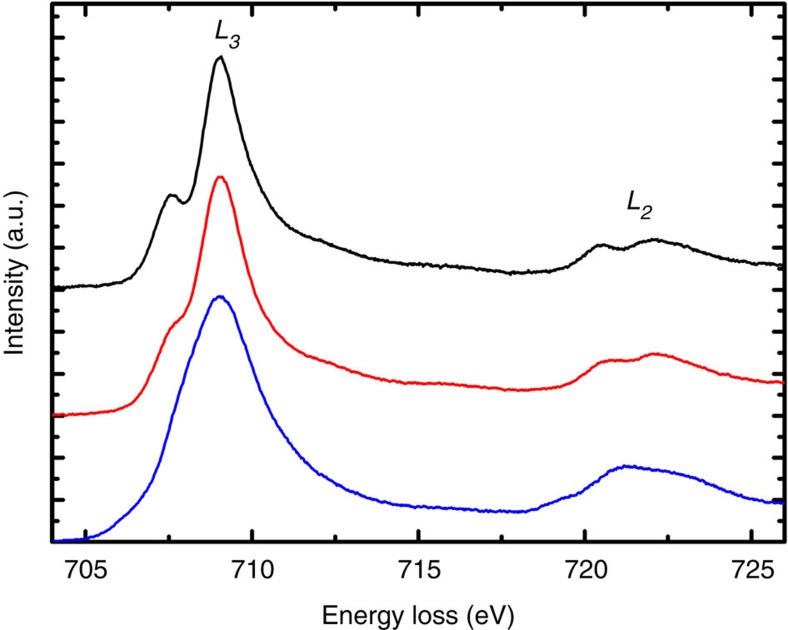
EEL spectra acquired from reference iron oxide samples. EELS analysis of the Fe 2*p L*_2,3_ edge experimentally acquired from pure samples of Fe_3_O_4_ (blue), γ-Fe_2_O_3_ (red) and α-Fe_2_O_3_ (black).

The bright-field TEM image of Fig. 1b shows the same Fe_3_O_4_ grain after exposure to 9 mbar O_2_ atmosphere at 700 °C for 8 h within the ETEM. Degradation of the surface of the grain is apparent, while the associated SAED pattern (Fig. 1b, inset) does not present any evidence for the formation of additional crystalline phases, with brightening of some planar reflections (for example, the −2 8 6 reflection) being attributable to slight tilting of the grain during annealing. However, the development of fine features in the associated EEL spectrum of the heated grain, taking the form of a small pre-peak in the *L*_3_ edge and post-peak in the *L*_2_ edge (Fig. 1c, red arrows), is indicative of a change in the Fe oxidation state towards γ-Fe_2_O_3_ or α-Fe_2_O_3_ (refs 15, 16, 17), as illustrated by the reference iron oxide EEL spectra displayed in Fig. 2. It is recognized that progressive oxidation induces the development of these small peaks, with complete oxidation to γ-Fe_2_O_3_ being associated with an ~1.3 eV splitting in the *L*_3_ edge^15^. Similarly, the Fe 2*p* edge in an α-Fe_2_O_3_ EEL spectrum is associated with a strong pre-peak located ~1.6 eV in front of the *L*_3_ edge^16^,^17^. Various iron hydroxides can also present similar pre-peaks in their Fe *L*_3_ edges; however, O_2_ is the only gas introduced into the system between acquisitions of EEL spectra, and hence the evolution of pre-peaks in this case is attributed solely to the effects of oxidation. The spacings between the central magnetic contours in the corresponding magnetic induction map (Fig. 1e), again flowing in a counterclockwise direction, were found to widen, most markedly towards the particle edge. Figure 1f,g presents magnetic contributions to the phase shifts used to construct Fig. 1d,e, respectively, and the reduction in amplitudes of the line profiles across their centres (dashed lines) is a strong indicator for loss of overall magnetic remanence in the Fe_3_O_4_ particle, as a consequence of oxidation (Fig. 1h, arrowed), again consistent with the progressive conversion of Fe_3_O_4_ towards γ-Fe_2_O_3_.

### Effect of oxidation on the direction of magnetization

**Figure 3:**
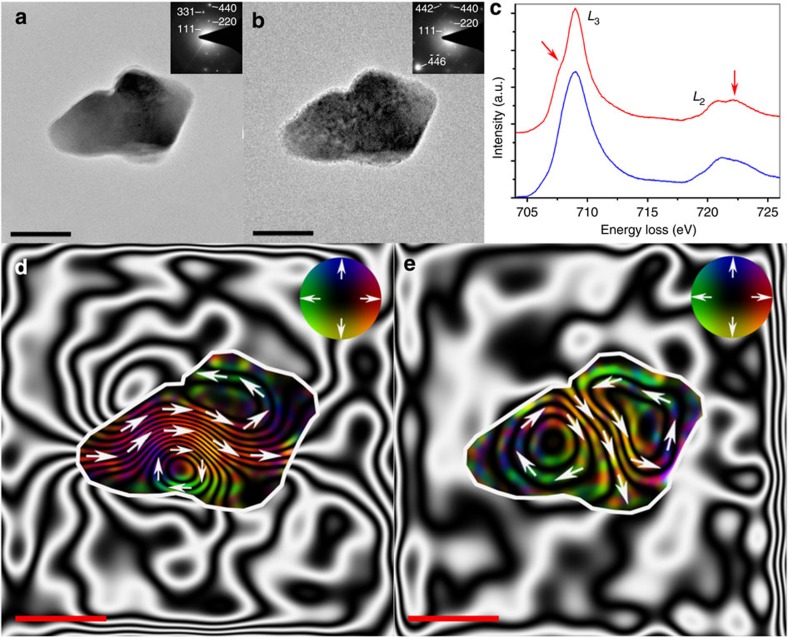
Visualized effect of oxidation on the magnetization of an elongated Fe_3_O_4_ particle. Bright-field TEM images acquired (**a**) before and (**b**) after *in situ* heating to 700 °C under 9 mbar of O_2_ for 8 h in an ETEM, with associated SAED patterns (inset) indexed to Fe_3_O_4_ (JCPDS No. 75–449). (**c**) Associated EEL spectra of the Fe 2*p L*_2,3_ edge acquired from the Fe_3_O_4_ particle before (blue) and after (red) annealing within the ETEM. Red arrows highlight the formation of pre- and post-peaks that indicate oxidation towards γ-Fe_2_O_3_. (**d**,**e**) Magnetic induction maps determined from the magnetic contribution to the phase shift reconstructed from holograms taken (**d**) before and (**e**) after *in situ* heating, revealing the PSD nature of the particle. The contour spacing is 0.20 radians for both magnetic induction maps. The magnetization direction is shown using arrows, as depicted in the colour wheel. Scale bars represent 100 nm.

Figure 3 illustrates the effect of accelerated oxidation on the magnetization of an elongated (~250 nm long, ~150 nm wide) Fe_3_O_4_ grain. The bright-field TEM image of Fig. 3a shows the grain morphology, while the associated SAED pattern (Fig. 3a, inset) indexes to Fe_3_O_4_, again supported by the complementary characteristic EEL spectrum of Fig. 3c. The corresponding magnetic induction map of Fig. 3d reveals closely spaced magnetic contours flowing from left to right through the elongated particle, interacting with a small vortex located at the bottom, along with a component of stray magnetic field, which is indicative of a PSD state.

The bright-field TEM image of Fig. 3b shows the same Fe_3_O_4_ grain after *in situ* heating in the ETEM at 700 °C under 9 mbar O_2_ atmosphere for 8 h. In a similar manner to the Fe_3_O_4_ grain shown in Fig. 1b, the elongated particle has degraded, while brightening of various planar reflections (for example, the −4 −4 6 and 4 4 2 reflections) in the associated SAED pattern (Fig. 3b, inset) was attributable to slight tilting of the particle during annealing. The additional development of a small pre-peak in the *L*_3_ edge and post-peak in the *L*_2_ edge of the corresponding EEL spectrum (Fig. 3c, red arrows) are again strong indicators for the effects of progressive oxidation towards γ-Fe_2_O_3_ or α-Fe_2_O_3_. The associated magnetic induction map (Fig. 3e) notably exhibits two vortices with widened magnetic contour spacings, flowing in opposite directions around a central transverse axis.

## Discussion

This combined ETEM and off-axis electron holography investigation has provided a visual representation of the effects of accelerated chemical oxidation on the magnetization (direction and intensity) of PSD Fe_3_O_4_ particles. Bright-field TEM imaging showed the native Fe_3_O_4_ particles to undergo degradation following *in situ* heating under an O_2_ atmosphere, while development of additional peaks in the Fe 2*p L*_2,3_ edges provides compelling evidence for the process of oxidation^12^, with the Fe 2*p L*_2,3_ edge acquired from the oxidized equiaxed Fe_3_O_4_ grain resembling that of pure γ-Fe_2_O_3_ (Fig. 2), as distinct from α-Fe_2_O_3_. Intriguingly, the SAED data from the oxidized Fe_3_O_4_ particles provided no evidence for the development of additional crystallographic reflections. This can be expected during the bulk conversion of an inverse spinel ferrite Fe_3_O_4_ towards the crystallographically similar Fe^2+^ cation-deficient γ-Fe_2_O_3_ phase, through topotactic transformation initiated at the surface, where faint extra spots characteristic of γ-Fe_2_O_3_ would only appear in the SAED pattern along specific zone axes.

The widening of the magnetic contours around the vortex in the oxidized elongated Fe_3_O_4_ grain (Fig. 1e) compared with its initial state, supported by line profile differences across the magnetic contributions to the phase shift (Fig. 1h), demonstrates that chemical alteration indeed leads to a loss of magnetization intensity. Clockwise rotation of the central magnetic contours from along the major axis of the elongated Fe_3_O_4_ particle (Fig. 3d) to a transverse axis (Fig. 3e) demonstrates the strong effect of oxidation on the magnetization direction, an effect previously considered to be dominated by shape anistropy^18^. In this case, the EEL peaks characteristic of γ-Fe_2_O_3_ in the Fe *2p L*_2,3_ edge of the oxidized elongated grain were notably less pronounced, in comparison with the equiaxed grain, suggesting that a significant proportion of Fe_3_O_4_ was still present. In this case, it is proposed that the tips of elongated grains are more susceptible to oxidation because of their larger exposed surface areas and shorter diffusion pathways. Preferential tip oxidation towards the less magnetic γ-Fe_2_O_3_ phase would alter the overall oxide phase distribution, with chemical modification of the elongated pure Fe_3_O_4_ grain to a PSD grain, containing a lower aspect ratio Fe_3_O_4_ core, prompting a shift towards lower energy and a more favourable direction of magnetization, the in-plane component of which is represented by the magnetic induction map of Fig. 3e. Indeed, magnetic domain states exist in three dimensions, and the shift in magnetization direction between the initial and oxidized elongated Fe_3_O_4_ grain suggests a greater degree of complexity associated with this process than can be fully accessed by two-dimensional (2D) in-plane representations of magnetization. Comparison between experimental and simulated magnetic induction maps, derived from multiphase three-dimensional (3D) micromagnetic models, could in future elucidate the 3D nature of the transformation observed in these magnetic domain states with chemical oxidation, providing fundamental insight into its effect on magnetic recording fidelity.

This combined ETEM and off-axis electron holography investigation has demonstrated changes in both remanent magnetic field strength and direction of magnetization within individual PSD particles as a consequence of accelerated chemical oxidation. When averaged over the millions of grains within a bulk palaeomagnetic sample, it is likely that the original directional information will be retained within the palaeomagnetic sample as not all grains will re-orientate their magnetization, although it is probable that some magnetic moments will re-align with the ambient field, giving rise to a CRM contribution to the total remanence. Certainly, the natural remanent magnetization intensity of a palaeomagnetic sample will most likely be decreased as a consequence of the oxidation process, resulting in an underestimate of ancient geomagnetic field intensity. Accordingly, palaeomagnetic data from samples showing evidence for chemical oxidation should be interpreted with care. The magnetic signal from natural rocks is inherently complex, comprising contributions from a mixture of low coercivity nonuniform MD magnetic grains, magnetically stronger nonuniform or vortex state PSD grains, and more stable high coercivity uniformly magnetized SD grains. As a step forward towards a better understanding of palaeomagnetic signals from the geomagnetic record, this study provides fundamental insight into the effect of chemical alteration on magnetic recording fidelity of the strongest magnetic mineral (magnetite) in the most commonly occurring domain state (PSD grains).

## Methods

### Sample details

Fe_3_O_4_ particles of diameter <200 nm (hydrothermally synthesized by Nanostructured and Amorphous Materials, USA) were cleaned with acetone and centrifuged for 6 min at 6,000 r.p.m. For the purpose of ETEM investigation, the particles were dispersed in distilled water using an ultrasonic bath before deposition on an Aduro E-chip TEM sample holder (Protochips, USA).

### Environmental TEM

Investigation of the *in situ* oxidation of Fe_3_O_4_ particles, under 9 mbar O_2_ atmosphere, at elevated temperature, established using a Protochips heating holder (room temperature to 700 °C at 1 °C s^−1^, as displayed by the Protochips temperature control), was performed using an FEI Titan E-Cell TEM with C_S_ corrector on the objective lens, operated at 300 kV. The high temperature of 700 °C was established to help compensate for the low pressure used during ETEM, being distinct from the ambient pressure conditions found in nature. EELS analysis was performed at a spectral resolution of ~0.3 eV, achieved through excitation of the monochromator, providing information on sample oxidation state. All TEM imaging and EEL spectra acquisition were performed on grains stabilized under high-vacuum conditions at ambient temperature, with *in situ* oxidation being performed in the absence of the electron beam to avoid any sample degradation through electron beam/material interaction during annealing.

### Magnetic imaging

Off-axis electron holograms were acquired at 300 kV using an FEI Titan 80–300 TEM in Lorentz mode, with a charge-coupled device camera and an electron biprism operated typically at 160 V. These experiments were performed at room temperature, with an acquisition time of 4 s. The total phase shift recorded using electron holography is sensitive to both the electrostatic potential and the in-plane component of the magnetic induction in the specimen. To isolate the magnetic contribution to the phase shift, the direction of magnetization in each particle was reversed *in situ* in the TEM by tilting the sample ±30° and turning on the conventional microscope objective lens to apply a magnetic field of 2 T to the sample, parallel to the direction of the electron beam. The objective lens was then turned off and the sample tilted back to 0° for hologram acquisition in field-free conditions (residual field <0.2 mT) with the particles at remanence. Following this procedure, holograms were recorded with the particles magnetized in opposite directions, while the mean inner potential was separated from the magnetic potential, as described by Dunin–Borkowski *et al*.^6^ For the construction of magnetic induction maps, the cosine of the magnetic contribution to the phase shift was amplified to produce magnetic phase contours. Colours were added to the contours to show the direction of the projected induction, as denoted by the colour wheels.

### Sample handling

In order to isolate the effects of chemical oxidation on the magnetization of the Fe_3_O_4_ grains, rather than possible temperature effects, samples were initially heated up to 700 °C within the Titan 80–300 TEM for 1 h and then cooled under vacuum, before acquisition of off-axis electron holograms from the native Fe_3_O_4_ grains. The Protochips TEM holder was then transferred to the Titan ETEM for the purpose of *in situ* chemical oxidation, with heating at 700 °C in a 9-mbar O_2_ atmosphere for 8 h, followed by imaging and EELS analysis under vacuum at ambient temperature conditions. The Protochips TEM holder and oxidized Fe_3_O_4_ grains were then transferred back to the Titan 80–300 TEM for acquisition of complementary off-axis electron holograms to appraise the magnetic response of the PSD grains.

## Author contributions

T.P.A. designed and carried out the experiments; A.R.M., W.W. and R.E.D.-B. conceived the study and supervised the research; T.K., T.W.H. and L.N. assisted with the experimental work and analysis; T.P.A. led the writing of the paper with contributions from A.R.M., P.D.B. and R.E.D.-B.

## Additional information

**How to cite this article**: Almeida, T. P. *et al*. Visualized effect of oxidation on magnetic recording fidelity in pseudo-single-domain magnetite particles. *Nat. Commun.* 5:5154 doi: 10.1038/ncomms6154 (2014).
